# Seizure attacks in viral encephalitis: influence on a course and outcome

**DOI:** 10.1186/cc9749

**Published:** 2011-03-11

**Authors:** E Rzadkiewicz, D Lipowski

**Affiliations:** 1 Medical University of Warsaw, Poland

## Introduction

Although occurrence of seizures is common in the course of viral encephalitis, its influence on outcome is less known [1].

## Methods

The frequency and type of seizures in 229 patients with viral encephalitis were studied. We compared frequency of loss of consciousness, mental disorders, respiratory failure, need for intubation, mechanical ventilation and hospitalization in the ICU, duration of hospitalization and degree of disability at discharge from the hospital according to the Glasgow Outcome Scale (GOS).

## Results

Patients with seizures (31), significantly more frequent in comparison with patients without attacks (198), presented: mental disorders in 17 (54.83%) versus 62 (31.31%) patients (*P *< 0.001), loss of consciousness in 28 (90.32%) versus 16 (8%) patients (*P *< 0.001) and need for intubation, mechanical ventilation and hospitalization in the ICU (34 versus 8 times, *P *< 0.001). The mean total time of hospitalization was substantially longer in patients with seizures in comparison with the group without them (24.43 vs. 15.9 days, *P *< 0.001). Patients presenting seizures were prognosticated worse in the scope of good recovery as well as every degree of disability in comparison with a group of patients without attacks (*P *= 0.001). Outcome after viral encephalitis according to GOS in patients with seizures (31) and without them (198) was as follows: GOS 5 (good recovery) - 19 (61.2%) versus 180 (90.9), GOS 4 (moderate disability) - 7 (22.5%) versus 12 (6%), GOS 3 (severe disability) - 4 (12.9%) versus 5 (2.5%), GOS 1 (death) - 1 (3.2%) versus 1 (0.5%).

## Conclusions

The occurrence of single generalized seizures, epilepsy and particularly status epilepticus had substantial influence on a course of viral encephalitis and worsened the outcome. Appearance of every type of seizure attack, independent of other clinical symptoms, was a good indicator of the disease severity.
